# Origin of anti-tumor activity of the cysteine-containing GO peptides and further optimization of their cytotoxic properties

**DOI:** 10.1038/srep40217

**Published:** 2017-01-16

**Authors:** Irina I. Tyuryaeva, Olga G. Lyublinskaya, Ivan S. Podkorytov, Nikolai R. Skrynnikov

**Affiliations:** 1 Institute of Cytology, Russian Academy of Sciences, St. Petersburg 194064, Russia; 2 Laboratory of Biomolecular NMR, St. Petersburg State University, St. Petersburg 199034, Russia; 3 Department of Intracellular Signaling and Transport, Institute of Cytology, Russian Academy of Sciences, St. Petersburg 194064, Russia; 4 Department of Chemistry, Purdue University, West Lafayette IN 47907, USA

## Abstract

Antitumor GO peptides have been designed as dimerization inhibitors of prominent oncoprotein mucin 1. In this study we demonstrate that activity of GO peptides is independent of the level of cellular expression of mucin 1. Furthermore, these peptides prove to be broadly cytotoxic, causing cell death also in normal cells such as dermal fibroblasts and endometrial mesenchymal stem cells. To explore molecular mechanism of their cytotoxicity, we have designed and tested a number of new peptide sequences containing the key CxC or CxxC motifs. Of note, these sequences bear no similarity to mucin 1 except that they also contain a pair of proximal cysteines. Several of the new peptides turned out to be significantly more potent than their GO prototypes. The results suggest that cytotoxicity of these peptides stems from their (moderate) activity as disulfide oxidoreductases. It is expected that such peptides, which we have termed DO peptides, are involved in disulfide-dithiol exchange reaction, resulting in formation of adventitious disulfide bridges in cell proteins. In turn, this leads to a partial loss of protein function and rapid onset of apoptosis. We anticipate that coupling DO sequences with tumor-homing transduction domains can create a potentially valuable new class of tumoricidal peptides.

The promise of peptide therapy has been recognized early on. With increasing knowledge on protein-protein interactions, it is often relatively easy to design a protein-binding peptide that would modulate protein function *in vitro*. However, the efficacy of such peptides *in vivo* is typically low because of the problems with targeting, membrane penetration, and poor proteolytic resistance. The success stories such as goserelin^1^ and liraglutide^2^ are usually associated with hormone-like peptides which rely on the existing receptor machinery to achieve high efficiency. Otherwise, to confront the limitations of peptide therapeutics, modified peptides have been extensively developed (e.g. peptides containing unnatural amino acids, peptides conjugated to other compounds, etc.). Ultimately, successful peptide ligands can be used as a basis to design small-molecule leads^3^. Generally, therapeutic peptides remain an important and steadily progressing area of pharmaceutical research.

Seven years ago, a new anti-cancer peptide GO-201 with the amino-acid sequence [**R**]_9_CQCRRKNYGQLDIFP has been introduced^4^. The sequence starts with nine arginines comprising a transduction domain, which facilitates the entry of the peptide into a cell^5^. The poly-arginine segment has been synthesized from unnatural D-amino acids with the intent to minimize potential proteolytic damage (hereafter one-letter codes of D-amino acids are typeset in bold). The remaining portion of the GO-201 sequence reproduces the fragment of the epithelial glycoprotein mucin 1, a prominent oncoprotein^6^. More specifically, GO-201 replicates a portion of the disordered cytoplasmic domain of mucin 1 (MUC1-CD). The notion behind this design is that GO-201 acts as a mimic of MUC1-CD capable of forming a dimer with a full-length protein^7^. Such dimer is expected to be non-functional and, therefore, GO-201 can be construed as an inhibitor of the MUC1-CD dimerization site.

GO-201 demonstrated activity against human breast carcinoma and prostate cancer cells, as established by cell culture experiments as well as experiments on animal models^4^,^8^. The activity against chronic myelogenous leukemia and pancreatic cancer has also been documented^9^,^10^. The investigators identified the key triplet of amino acids, CQC, that proved to be responsible for the anti-tumor activity of GO-201. A control peptide CP-1 containing two alanine-for-cysteine substitutions, [**R**]_9_AQARRKNYGQLDIFP, showed no appreciable cytotoxic properties. It is understood that the special role of cysteines is due to their ability to form disulfide bonds^11^.

Concurrently with GO-201, a shorter version of the peptide, [**R**]_9_CQCRRKN, has been introduced under the name GO-202. The length of the mucin-derived sequence in this variant of the peptide is only seven residues. Generally, it is difficult to expect that 7-residue segment would retain a high degree of selectivity against the target which is disordered (MUC1-CD). Nevertheless, GO-202 showed the level of activity identical to GO-201 when applied to acute myeloid leukemia and lung adenocarcinoma cells^12^,^13^. Soon thereafter an altered version of GO-202 was tested, which had the same sequence but was synthesized from all right-handed D-amino acids, [**R**]_9_**CQCRRKN**. The rationale was to further improve the proteolytic resistance of the peptide. This variant, which was branded GO-203, showed high level of activity against non-small cell lung cancer, prostate cancer, acute myeloid leukemia, breast cancer, multiple myeloma, and other forms of cancer^14^,^15^,^16^,^17^,^18^,^19^,^20^. Phase I clinical trials of GO-203 in patients with advanced solid tumors (including lymphomas) have been completed. Phase I/II trials in patients with relapsed or refractory acute myeloid leukemia are currently underway and further trials are planned for patients with multiple myeloma (clinicaltrials.gov identifiers NCT02204085 and NCT02658396, respectively).

In contemplating these results, we were particularly intrigued by the fact that GO-203, which is comprised of all D-amino acids, displayed a high level of anti-tumor efficacy. Indeed, GO-201 and 202 carry a stretch of L-amino acids replicating a segment from MUC1-CD. Therefore, they can be conceivably viewed as mimics of MUC1-CD that are capable of dimerizing with the full-length protein and thus preventing formation of the functional homodimer. Conversely, GO-203, which is comprised of D-amino acids, has a fundamentally different topology (corresponding to a mirror image of the respective MUC1-CD fragment). From a structural standpoint, GO-203 has little in common with MUC1-CD. Hence there is no reason to expect that GO-203 should show any significant preference for MUC1-CD as a potential binding target. This led us to hypothesize that the origin of anti-tumor activity of GO-203, as well as other GO peptides, is actually unrelated to MUC1-CD but rather lies elsewhere.

This hypothesis led us to further important considerations. Originally, GO peptides have been designed to replicate the sequence of MUC1-CD. However, if true mechanism of their activity is unrelated to mucin, then the choice of the sequence does not need to be limited to mucin fragments. In other words, there is an opportunity to optimize the sequence of GO peptides such as to further enhance their tumor-suppressor properties. In doing so it is necessary to retain the pair of closely spaced cysteines, which are key to the peptide activity. Other residues can be varied (either in a combinatorial fashion or using a rational design strategy) to achieve maximum anti-tumor activity.

In pursuing this agenda, we have conducted a series of experiments on different cell lines employing GO peptides and a number of new peptides carrying two proximal cysteines. Our main findings are summarized below: (i) GO peptides are broadly cytotoxic – they can be lethal not only to cancer cells, but also to normal cells; (ii) The cytotoxic effect of GO peptides is unrelated to the expression of mucin – in fact, the cell line that strongly overexpresses MUC1 turns out to be less vulnerable to the effect of GO peptides, whereas other cell lines that show low-level MUC1 expression prove to be extremely sensitive; (iii) We designed several new cysteine-containing peptides which showed considerably stronger cytotoxic activity than the original GO peptides, while bearing no resemblance to MUC1.

The obtained results and, in particular, the results of the assays involving newly designed peptides led us to formulate a new hypothesis regarding the mechanism of the observed peptide activity. We propose that the potent peptides possess some degree of disulfide oxidoreductase activity. In other words, they are involved in dithiol-disulfide exchange reactions which promote disulfide bonding and cross-linking in cytosolic proteins. In turn, this leads to a loss of protein function and rapid apoptotic demise of the treated cells. This hypothesis is directly supported by the observation that GO-202 shows properties of (moderately effective) disulfide oxidoreductase in our experiment on oxidative refolding of lysozyme. We propose the term “DO peptides” to describe a broad category of cysteine-containing peptides with the expected disulfide oxidoreductase (DO) activity. In the concluding section of this paper we discuss potential options to make DO peptides useful in the context of cancer therapy.

## Results

### Cytotoxic activity of GO-202 and GO-203

In this section we examine certain basic facts about the cytotoxic effect of GO peptides. We focus on GO-202 where the active sequence is synthesized from L-amino acids, allowing one to draw a connection to native protein sequences (see below). The corresponding control peptide CP-2 has also been included in the tests. In addition, we also employed GO-203, which shows maximum efficiency against cancer cells.

**Figure 1:**
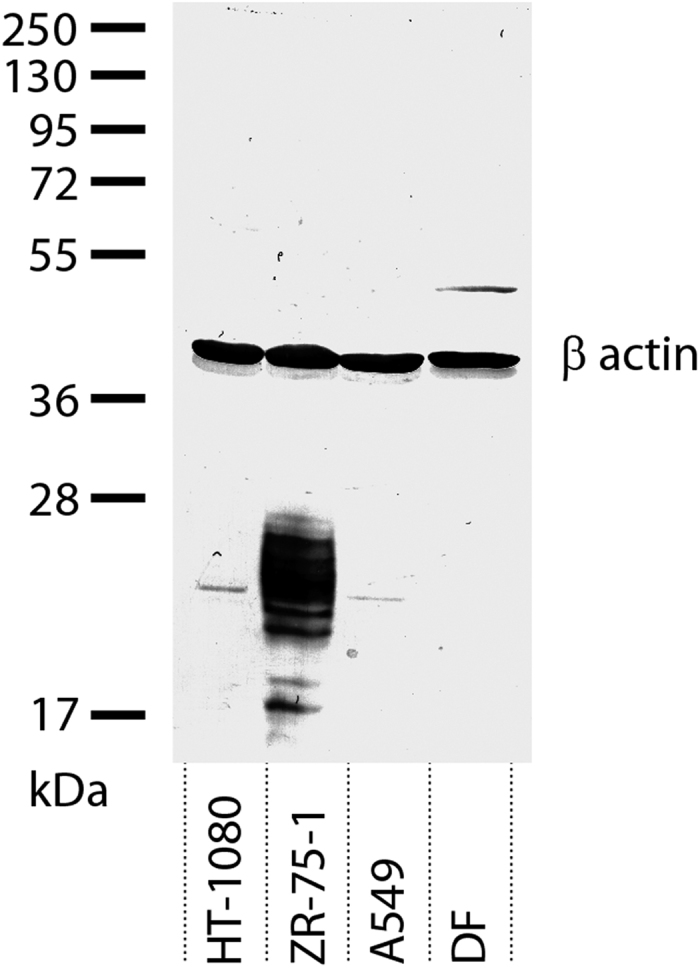
Western blot analysis of mucin 1 expression in four different cell lines. The image has been overexposed to reveal the weaker bands (see Fig. S1 for the low-exposure image).

At this initial stage we choose to conduct the tests on human breast carcinoma cell line ZR-75-1, human fibrosarcoma cell line НТ-1080, human lung carcinoma cell line А549, and the primary culture of human dermal fibroblasts (DF). To probe the status of these cells with respect to MUC1 expression we have performed a Western blot analysis using an antibody against MUC1-CD. The results are presented in Fig. 1. In agreement with the prior literature data, mucin 1 is seen to be strongly overexpressed in ZR-75-1^21^,^22^. The fine structure of the corresponding band (lane 2) is indicative of variable glycosylation pattern typical of MUC1 in breast carcinomas^23^. On the other hand, HT-1080 and A549 both show low level of MUC1 expression (lanes 1 and 3) consistent with the previously reported results^22^,^24^. Likewise mucin 1 is weakly expressed in dermal fibroblasts (lane 4), in agreement with the recent findings^25^. To explain the unusual mass of the detected fragment one should consider two factors: (i) MUC1 displays much higher level of glycosylation in normal cells compared to cancer cells, which has a strong effect on its electrophoretic mobility, and (ii) there are many splicing isoforms of MUC1, including those where (due to the disruption or deletion of the SEA domain) MUC1 consists of one long chain^26^. Note that these variations occur in the extracellular domain of MUC1 and have no influence on the cytoplasmic domain, which is recognized by the primary antibody. In summary, the results shown in Fig. 1 confirm the existing knowledge that ZR-75-1 strongly overexpresses mucin 1, while the other three cell types show much lower level of expression.

**Figure 2:**
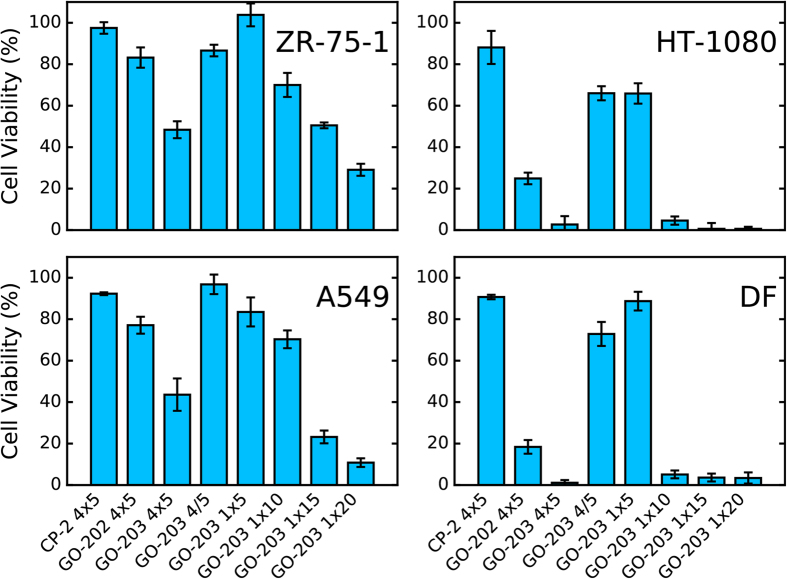
Cell viability under treatment by CP-2 (control), GO-202, and GO-203 peptides for cell cultures of ZR-75-1, HT-1080, A549, and dermal fibroblasts (DF). The treatment protocols are: 4 × 5 – four daily doses of 5 μM delivered without exchanging the growth medium; 4/5 – four daily doses of 5 μM delivered with exchanged growth medium; 1 × 5, 1 × 10, 1 × 15, and 1 × 20 – a single dose of 5, 10, 15, or 20 μM, respectively. MTS reagent was added to the wells 96 h after the first (and sometimes the only) application of the peptide and the optical density at 492 nm was measured to determine the fraction of the surviving cells.

As a next step, we used MTS assay^27^ to examine the effect of GO peptides on the viability of the four described cell cultures as a function of different treatment schemes. The results are summarized in Fig. 2. First, we have applied the protocol that is similar to the one previously employed in all studies of GO peptides: 5 μM of peptide has been added daily to the growth medium for 4 days without exchanging the medium^4^,^14^. This treatment regimen is labeled 4 × 5 underneath the graph. Alternatively, we introduced 5 μM of peptide daily while exchanging the medium (denoted 4/5). Unlike the previous protocol, it avoids potential build-up of the peptide in the medium. Finally, we have tested the most basic scheme involving one-time dose of the peptide with either 5, 10, 15, or 20 μM concentration (labeled 1 × 5, 1 × 10, 1 × 15, and 1 × 20, respectively).

The most striking result from this assay, as becomes evident from Fig. 2, is that the efficacy of GO peptides is unrelated to the level of MUC1 expression. Indeed, the cell line ZR-75-1, which strongly overexpresses mucin 1, proves to be largely resistant to GO-202 and -203. On the other hand, HT-1080 and dermal fibroblasts are both highly susceptible despite their low level of mucin 1 expression (in fact, HT-1080 was initially described as “non-expressing” line ref. 22). Another cell line with low-level MUC1 expression, A549, proves to be relatively resistant, with response profile similar to ZR-75-1. These observations fit well with our original line of reasoning where we argue that all-D-amino-acid peptide GO-203 cannot act as a selective inhibitor of mucin dimerization (see *Introduction*). The mechanism of its activity must lie elsewhere.

No less important is the observation that GO peptides are lethal not only to cancer cells, but also to normal cells. As shown in Fig. 2, the treatment of dermal fibroblasts, which are normal non-cancerous cells, results in cell death *on par* with the most susceptible cancer line HT-1080. Another example of this behavior, involving endometrial mesenchymal stem cells, is shown in what follows. We conclude that GO peptides should properly be described as cytotoxic peptides rather than antitumor peptides. This finding fits well with what is generally known about GO peptides. Indeed, the activity of GO-type peptides is critically dependent on two closely spaced cysteines. In fact, the activity is preserved – or even enhanced – upon substitution of amino acids other than cysteines (see below). Clearly, a pair of cysteine residues in a short disordered peptide cannot constitute a sound basis for target selection, i.e. this is insufficient to discriminate between cancer and non-cancer cells. In the light of this result, it is also unsurprising that GO peptides have demonstrated activity against a multitude of different cancer types.

Finally, Fig. 2 offers some important information concerning the efficacy of different peptides and treatment regimes. The control peptide CP-2, where both cysteine residues are replaced with alanine, shows only minimal effect compared to the untreated culture (first bar in each panel). The same treatment scheme using daily additions of GO-202 has significant impact on HT-1080 and dermal fibroblasts (second bar). Application of GO-203 leads to essentially complete cell death in these two cultures, while ZR-75-1 and A549 suffer considerable damage (third bar). As already noted, the increased efficiency of GO-203 relative to GO-202 can be attributed to its improved stability.

Of particular note, there is a pronounced difference between 4 × 5 regime, corresponding to cumulative daily additions (third bar), and 4/5 regime, corresponding to non-cumulative additions (fourth bar). The latter scheme proves to be inefficient for all of the analyzed cell cultures. In other words, repeated exposure to 5 μM doses does not cause any significant amount of cell death. It is apparently the build-up of GO-203 concentration in the growth medium occurring in the 4 × 5 protocol that leads to cell death. In this sense the schemes such as 4 × 5, which have been traditionally used for GO peptides, are problematic because one cannot easily quantify the build-up of the peptide concentration in the media (note that such build-up is itself a function of cell death, which makes an analysis needlessly complicated).

We have also tested the standard protocol involving one-time treatment: 1 × 5, 1 × 10, 1 × 15, and 1 × 20. A single application of 5 μM dose proves to be essentially inconsequential (fifth bar in all panels of Fig. 2), consistent with the above discussion. However 10 μM dose nearly wipes out HT-1080 cells and skin fibroblasts; the population does not recover after 96 h, when the reading of the optical density is taken. Increasing the dose to 20 μM makes GO-203 substantially cytotoxic also for ZR-75-1 and A549 (rightmost bar). These results lead us to conclude that one-time treatment using an appropriate dose of peptide is a suitable protocol to further investigate the mechanism of activity for GO peptides and their more potent derivatives.

### Observation of apoptosis in cell lines with different sensitivity to GO-203

**Figure 3:**
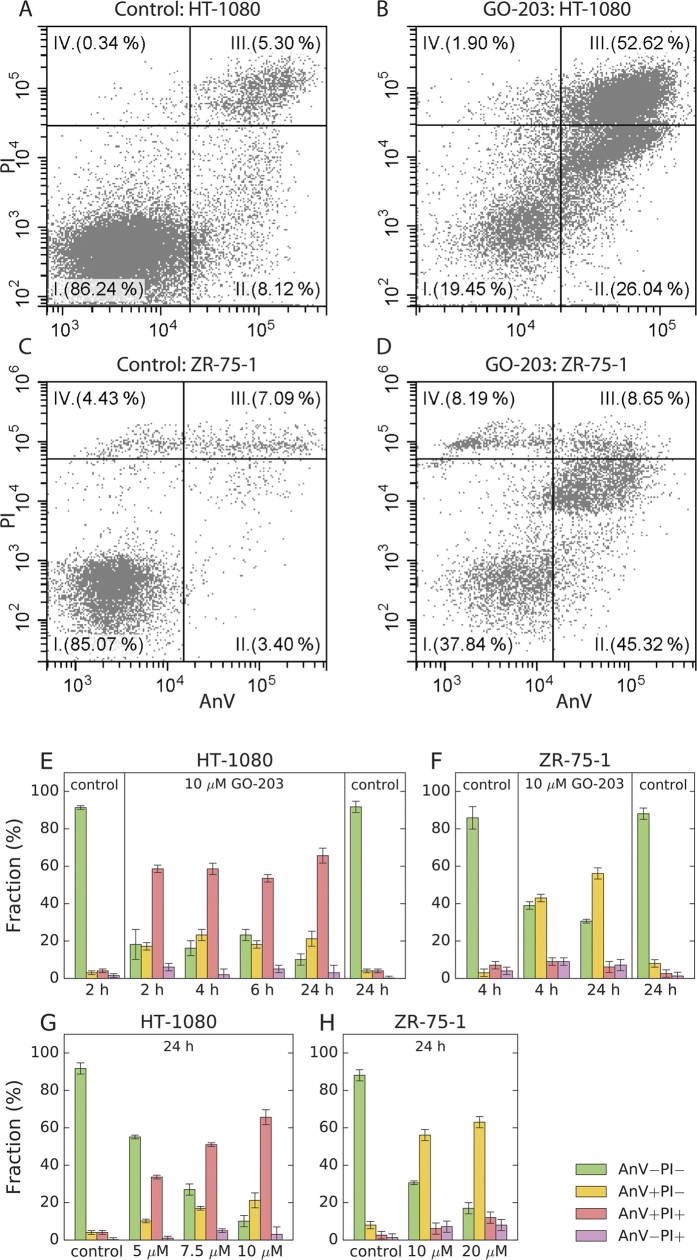
GO-203-induced apoptosis in HT-1080 and ZR-75-1 cells as assessed by FITC Annexin V (AnV) and propidium iodide (PI) double staining. (**A–D**) Flow cytometry histograms for the cells incubated for 4 h with 10 μM of GO-203 and the untreated (control) cells. (**E–H**) Apoptotic fractions in the GO-203-treated and untreated (control) cells as a function of incubation time and GO-203 concentration; color coding is explained in the legend.

The apoptotic mode of cell death following GO-203 treatment has been demonstrated by flow cytometry using FITC Annexin V assay with propidium iodide as a vital dye. The representative dot plots are shown in Fig. 3A–D; the fractions of apoptotic cells, evaluated as a function of peptide concentration and incubation time, are quantified in Fig. 3E–H. For the cell line HT-1080, which is highly sensitive to the cytotoxic effect of GO-203, the apoptosis occurs sufficiently rapidly. Already 2 h after administration of 10 μM dose, the system reaches a steady state with 18% of healthy cells, 17% of early apoptotic cells, and 58% of late apoptotic cells. It remains in this state for the entire period of observations, 24 h.

Similarly fast apoptotic process is observed in ZR-75-1, although this cell line is less susceptible to the cytotoxic effect of GO-203. 4 h after administration of 10 μM dose, 39% of the cells remain healthy, 43% undergo early apoptosis, and 9% are in the late apoptosis stage. This proportion remains unchanged after 24 h. The fact that GO-203-induced apoptosis occurs rapidly in both sensitive and relatively insensitive cell lines is relevant in the context of its cytotoxic mechanism (discussed later in the text).

Also illustrated in Fig. 3G,H is the dose-dependent response of the treated cells. In HT-1080, we observe a clear-cut trend whereby the fraction of the healthy cells declines with the increase in peptide dosage, while the fraction of the early- and especially late-apoptotic cells shows progressive growth. In ZR-75-1, the healthy cells are gradually turned into early apoptotic cells; however, even when treated with 20 μM GO-203 a large proportion of the cells remains viable. The fraction of the late apoptotic cells is consistently small and comparable to that seen in the control sample. It is possible that apoptotic ZR-75-1 cells in quadrant II undergo disintegration instead of moving to quadrant III, Fig. 3D. Importantly, the Annexin V data are consistent with the results of the MTS assay which registers limited damage to the ZR-75-1 cells, see Fig. 2.

**Figure 4:**
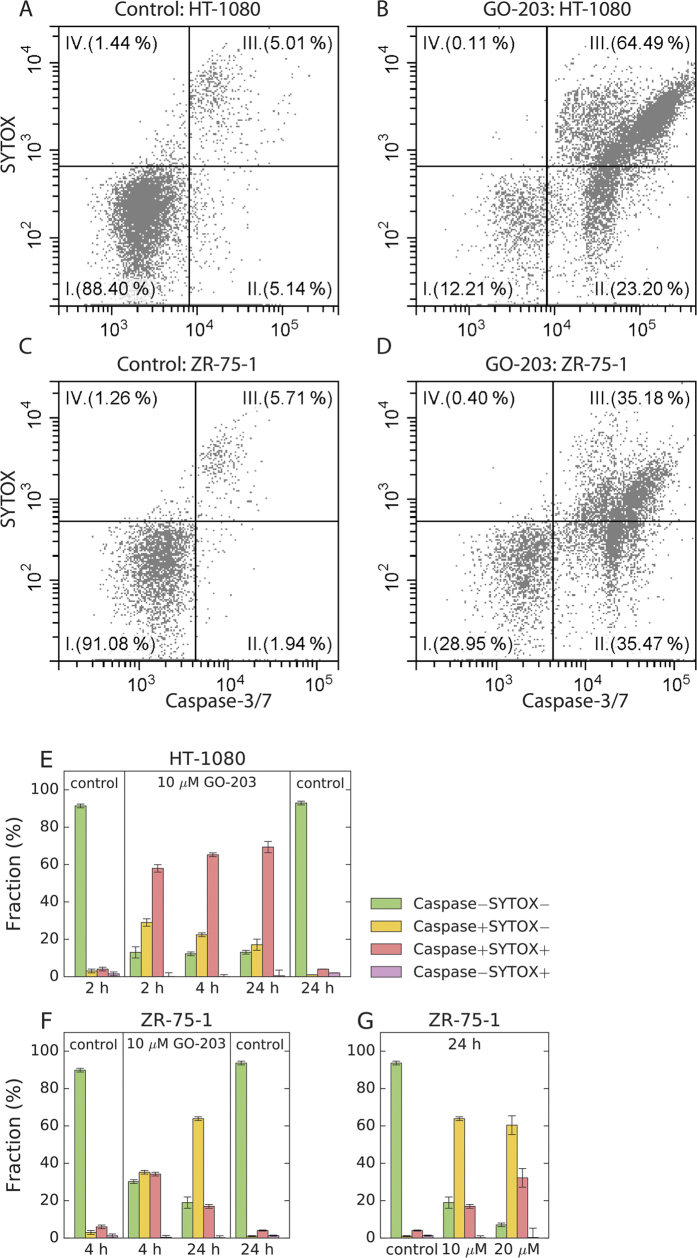
GO-203-induced apoptosis in HT-1080 and ZR-75-1 cells as assessed by the double staining with CellEvent caspase-3/7 green detection reagent and vital dye SYTOX. (**A–D**) Flow cytometry histograms for the cells incubated for 4 h with 10 μM of GO-203 and the untreated (control) cells. (**E–G**) Apoptotic fractions in the GO-203-treated and untreated (control) cells as a function of incubation time and GO-203 concentration.

A complementary perspective on GO-203-induced apoptosis is provided by the caspase 3/7 activation assay, Fig. 4. In both HT-1080 and ZR-75-1, the data offer a clear view of the early and late apoptotic populations. The time dependence illustrated in Fig. 4E,F confirms that the onset of apoptosis occurs sufficiently rapidly in both cell lines – by the time of flow-cytometry observations the system has already approached the steady state. In the literature there is some evidence that activation of executioner caspases may precede the translocation of phosphatidylserine to the outer leaf of the membrane^28^,^29^. While this could be in principle relevant for comparison between the Annexin V assay and caspase activation assay, one should bear in mind that in our case both assays offer a snapshot of a fairly advanced apoptotic process. As discussed below, the apoptosis is in fact triggered within the first hour after the peptide treatment.

**Figure 5:**
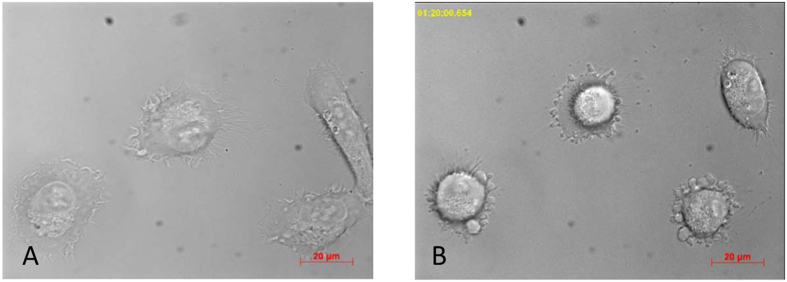
Time-lapse microscopy of HT-1080 cells. (**A**) Cells before the addition of the peptide and (**B**) 1 h 20 min after the addition of 5 μM GO-203.

**Figure 6:**
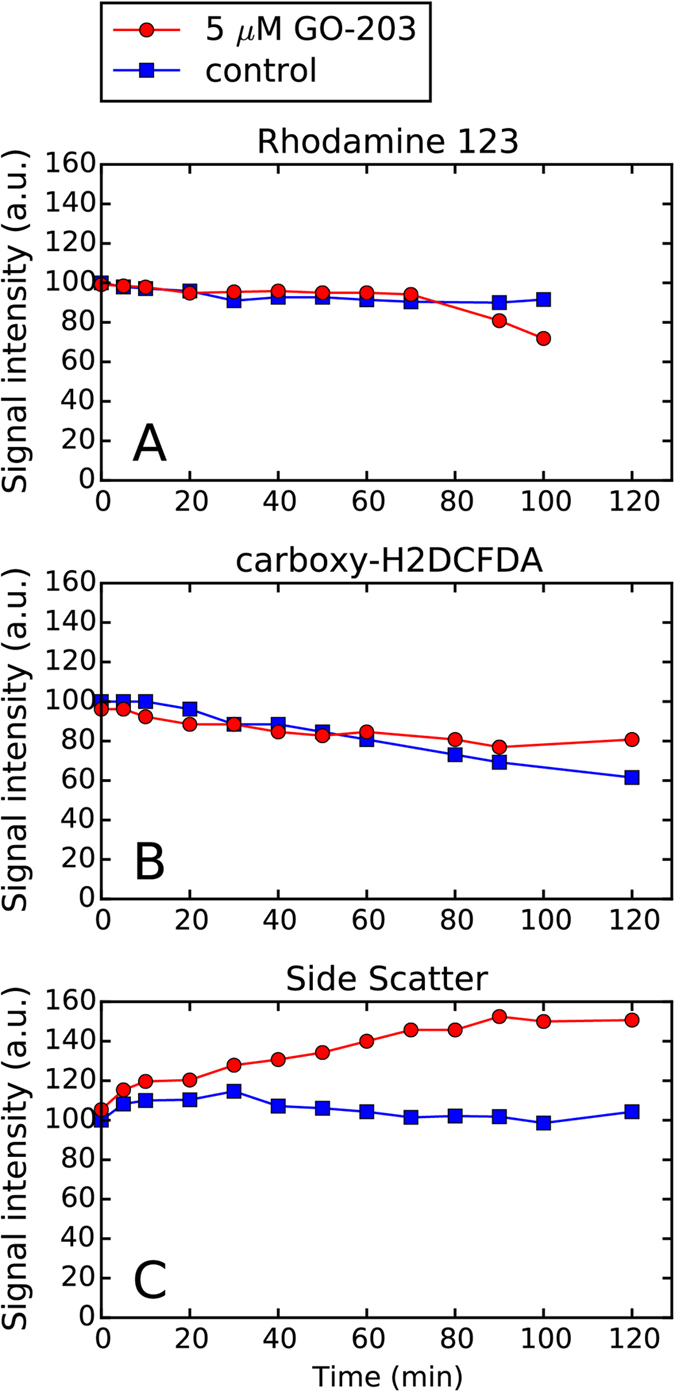
Dynamics of (**A**) mitochondrial membrane potential, (**B**) intracellular ROS levels, and (**C**) cell refractive properties in suspended HT-1080 cells following the addition of 5 μM GO-203.

The data from time-lapse microscopy on HT-1080 cells provide a visual evidence of GO-203-induced apoptosis, Fig. 5 (see also Film S1). The morphological features of apoptosis – cell contraction (rounding) and consequently membrane blebbing – manifest themselves within the first hour after addition of the peptide. After 1.5 h we observe the fully developed picture of apoptosis. This is illustrated in the right panel of Fig. 5: three cells in this photograph experience blebbing, while the fourth cell located in the upper right portion of the image has undergone detachment and rounded up (it is soon to enter the blebbing phase). This time line is confirmed by the side angle light scatter data from the flow-cytometry experiment, Fig. 6C. As can be seen in this graph, the scatter starts to rise (i.e. diverges from the control curve) 30 min after application of GO-203. This behavior reflects the onset of dynamic blebbing and the concomitant increase in the roughness of the cell surface. In this context it is worth noting that actomyosin cortex contraction and blebbing are subsequent to (and in fact driven by) caspase activation^30^. The condensation of nucleus and cytoplasm in apoptotic cells can also contribute to the increased side scatter^31^.

In summary, we present here several lines of evidence indicating that GO-203 induces apoptotic death in both HT-1080 and ZR-75-1 cells. The onset and the progression of the apoptosis are relatively fast, on the time scale of 1 h. For the rapid process like this we do not expect that the cytotoxic effect of GO-203 has to do with cell cycle progression. Indeed, the cell cycle phase distribution remains unaffected by addition of the peptide. For example, in the untreated (control) HT-1080 sample the distribution between G_0_/G_1_, S, and G_2_/M phases is 52%, 23% and 26%, respectively. After 24 h incubation with 5 μM GO-203 this proportion is essentially unchanged at 50%, 24%, and 27%. Similar results have also been obtained for ZR-75-1 cell line (see Fig. S2).

### Influence of GO-203 on mitochondrial membrane potential and cellular ROS level

In broad terms, apoptosis is classified into two fundamental modes: mitochondrial pathway and death receptor pathway. In the former mechanism, apoptotic signals lead to a loss of mitochondrial membrane potential, termination of mitochondrial ATP synthesis, overproduction of reactive oxygen species (ROS) and their release into cytosol along with a number of pro-apoptotic proteins that are normally confined to the intermembrane space of the mitochondria^32^. To determine whether this mechanism is relevant for GO-203-induced cell death, we have analyzed the time variation of mitochondrial membrane potential and cellular level of ROS over the time interval of 2 h following the addition of the peptide. Toward this goal, the cells were preloaded with Rhodamine 123 or carboxy-H2DCFDA fluorescent dyes, then washed, suspended in the growth medium, and treated with GO-203. The fluorescence was measured concurrently with the side angle light scatter.

Figure 6A shows the Rhodamine 123 fluorescence intensity in HT-1080 cells subjected to one-time 5 μM dose of GO-203. As can be seen from this figure, the mitochondrial retention of Rhodamine 123 by the treated cells remains the same as in the control cells for at least 70 min after the addition of the peptide (cf. red and blue profiles in Fig. 6A). Only at 90–100 min we observe a moderate decrease of the fluorescence signal from the treated cells, which indicates the drop in mitochondrial membrane potential. As discussed above, the onset of apoptosis in HT-1080 occurs considerably earlier than that. Of particular note, the side-scatter data in Fig. 6C, which have been recorded concurrently with the fluorescence data, suggest that dynamic blebbing begins in this system already after 30 min. Therefore we assume that mitochondrial membrane permeabilization (MMP) occurs relatively late in the game and is unlikely to be a key element of the GO-203-induced apoptosis.

To confirm this finding we have also used the carboxy-H2DCFDA probe of the general oxidative stress. As indicated above, the loss of mitochondrial membrane integrity is accompanied by a massive release of ROS into cytosol, which offers an additional possibility to detect MMP. The results of the ROS assay in HT-1080, where the intensity of fluorescence signal reflects the level of intracellular oxidants, are shown in Fig. 6B. In this graph, the red curve (treated cells) begins to diverge from the blue curve (control) at 80–90 min. Finally, after 120 min there is evidence that treated cells suffer from the elevated level of ROS. These results are consistent with the Rhodamine 123 assay (see above). Together they indicate that mitochondria is compromised relatively late during the execution phase of the apoptosis and, therefore, the MMP mechanism cannot be viewed as a driving force of the cell death.

Generally, the role of ROS in apoptosis is not limited to the MMP and its consequences. There are multiple pathways whereby oxidative stress can trigger apoptosis, including the MMP-mediated apoptosis, e.g. through JNK signaling, FLIP_L_ signaling, or perturbing the GSH/GSSG balance^33^,^34^,^35^. The above-described assays provide no evidence of a ROS insult that is prerequisite for any such mechanism.

The experiment using cabroxy-H2DCFDA has been designed to detect possible early surge in the ROS level (none has been detected). The limitation of this experiment is that it is essentially a one-sided test – it can register an increase, but not a decrease in the level of ROS (any decrease in fluorescence in our experimental scheme should be attributed to the leakage of the fluorescent carboxy-DCF ions from the cell). To address this deficiency, we have conducted a series of additional ROS measurements (see Fig. S3).

First, we have carried out a control experiment using ROS-insensitive DCFDA dye. In this experiment, we added 5 μM GO-203 to the dish containing HT-1080 cells, then incubated the cells with DCFDA dye, washed them, suspended in the buffer, and measured the fluorescence. As it turns out, the intensity of fluorescence 30 min after the addition of the peptide is identical to that from the control cells. This means that (i) the activity of esterases responsible for conversion of non-fluorescent DCFDA probe into fluorescent DCF does not change in response to GO-203 treatment and (ii) the rate of DCF leakage through the cell membrane also remains unaffected.

Having established these premises, we repeated the measurements using the ROS-sensitive probe H2DCFDA instead of DCFDA. We have observed small, but statistically significant difference between the HT-1080 cells treated by GO-203 and control cells. Specifically, 30 min after application of the peptide the fluorescence signal from the treated cells was ca. 20% lower than the control, 82 ± 6 vs. 100 ± 7 (arbitrary units). The drop in fluorescence from cells that have been treated with GO-203 should be unequivocally attributed to the decreased level of ROS. The effect is small and unlikely to be the source of GO-203 cytotoxic activity. However, as described in the *Discussion* section, it is consistent with the proposed mechanism of cytotoxicity. Similar H2DCFDA/DCFDA measurements were also conducted on ZR-75-1 cell line. The detected drop in ROS level was below the level of statistical significance, 98 ± 3 vs. 100 ± 11 (see Fig. S3). Note that ZR-75-1 is intrinsically less sensitive to the cytotoxic effect of GO-203 than HT-1080.

Finally, the same experimental scheme as described above for H2DCFDA/DCFDA measurements was also used with Rhodamine 123. The results for HT-1080 cells were in agreement with the data shown in Fig. 6, confirming that mitochondrial membrane potential is fully maintained 30 min after the application of GO-203. The same observation has been made for ZR-75-1.

### Peptides with altered sequence show stronger activity

The key role of the two cysteine residues in the GO peptides has been established early on ref. 4. At the same time, here we have shown that the antitumor (or, more accurately, cytotoxic) effect of the GO peptide is likely unrelated to the epithelial glycoprotein MUC1. Therefore there is no logical reason why the amino-acid sequence of the GO peptides should replicate the fragment of MUC1-CD. We predict that the sequence can be altered leading to new peptides with a similar or even higher level of activity. In designing such new sequences, one needs to retain a pair of closely spaced cysteines which are the key to the cytotoxic effect. Also the [**R**]_9_ or another similar transduction domain is needed. Other than that the amino-acid sequence can be varied.

Based on this realization, our agenda here is to alter the peptide sequence such as to enhance its antitumor (cytotoxic) activity. The objective is two-fold. First, by re-engineering the peptide we can shed light on the true mechanism of its cytotoxic activity. Second, the optimized sequence can be fused with a tumor-homing transduction domain^36^ or otherwise adapted for targeted delivery, which should allow us to evaluate its true therapeutic potential.

There are two possible strategies to re-design GO peptide. One is the combinatorial approach where many possible combinations of amino-acids are tested in a more or less systematic fashion. If this strategy is implemented in a high-throughput manner, it can lead to a highly optimized peptide. At the same time, this route is expensive and unlikely to shed light on the origin of the peptide cytotoxicity. Another option is a rational design strategy, where the choices are made based on some sort of prior knowledge. This strategy may not lead to the highly optimized product, but has the advantage of being more affordable and potentially more informative with regard to the peptide mechanism of activity. It is this latter approach which is pursued in our study.

The new peptides tested in our work are listed in Table 1. First we focus on the peptides containing CxC and CxxC motifs in the context of their well-documented disulfide oxidoreductase properties. As mentioned above, these motifs are involved in dithiol-disulfide exchange reactions. The efficiency of commonly known disulfide oxidoreductases depends on multiple factors such as redox potential, thiol pKa, and others. The Cx(x)C sites with less negative redox potential (e.g. −120 mV in the case of periplasmic factor DsbA^37^) are characteristic of disulfide oxidases; they catalyze the formation of disulfide bridges in proteins. Conversely the Cx(x)C sites with more negative redox potential (e.g. −270 mV for bacterial thioredoxin^38^) are characteristic of disulfide reductases; they catalyze the dissolution of disulfide bridges. Finally, the Cx(x)C sites with intermediate redox potential are typical of disulfide isomerases; they can catalyze both formation and dissolution of disulfide bonds. Another significant factor that influences the enzymatic activity is the thiol pKa. Indeed, it is known that thiolate is much more reactive than thiol in the context of the dithiol-disulfide exchange reaction^39^. Other factors, such as steric interactions at the active site, are relevant as well.

One instructive example of the redox-active peptides in Table 1 is the peptide labeled DIL (disulfide-isomerase-like). Originally, Woycechowsky and Raines have investigated the CGC peptide, where they measured the redox potential of −167 mV^40^. As expected, this peptide showed a certain level of disulfide isomerase activity. Recently, a longer peptide, RKCGCFF, has been introduced by Liu *et al*.^41^. The two basic residues, RK, have been added to the sequence with the intent to lower the thiol pKa values, while the two hydrophobic residues, FF, have been included to improve binding of the peptide to various protein targets. The peptide showed increased efficiency as a mimic of disulfide isomerase, facilitating oxidative refolding of lysozyme *in vitro*. We took advantage of these results to design a cell-penetrating peptide with disulfide isomerase properties. Toward this goal we have combined RKCGCFF fragment with the [**R**]_9_ leader sequence, arriving at the peptide that has been termed DIL.

In addition to the DIL and other redox-active peptides, we also tested peptides with a different presumed mechanism of activity. For instance, we considered a possibility that two or more GO peptides may collectively coordinate and sequester metal ions inside the cell. To test this hypothetical possibility, we have used the sequence extracted from the copper-inducible repressor of the cop operon in *E. hirae*. This sequence contains not one, but two CxC sites that can together bind copper^42^. The standard [**R**]_9_ poly-arginine tag has been prepended to this sequence, leading to the CopYL peptide (see Table 1).

**Figure 7:**
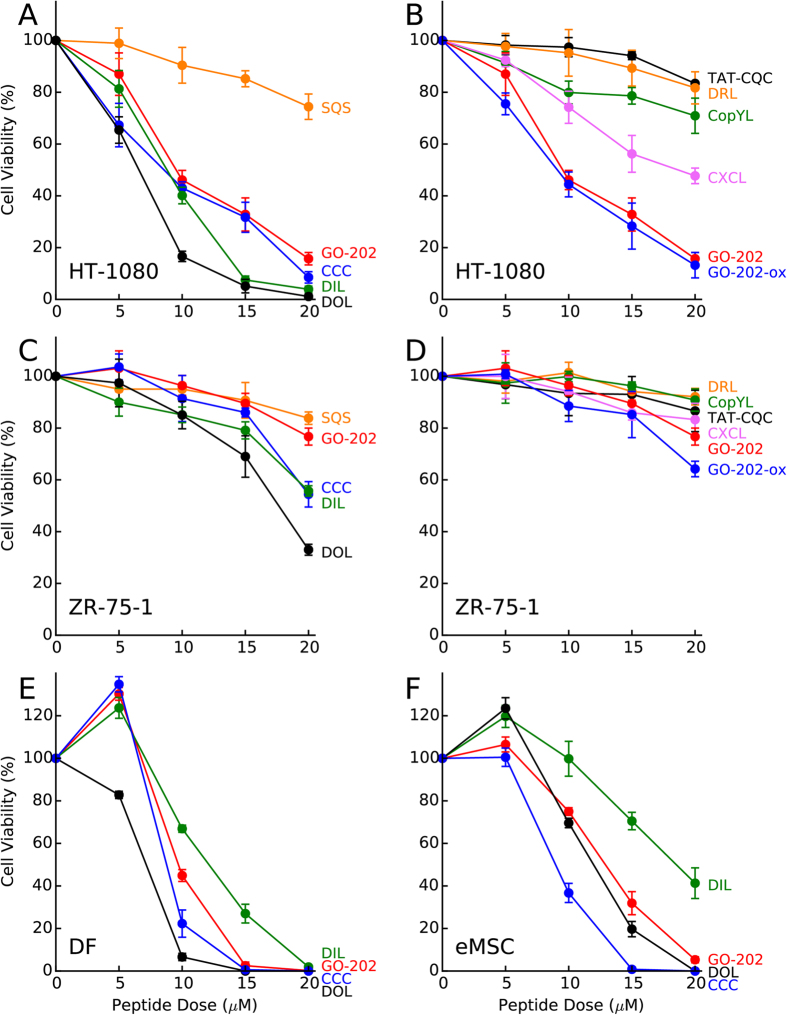
Dose-response data for different cell cultures treated by GO-202 and 9 other peptides (see Table 1). The data are according to the MTS assay, where the optical density was measured 96 h after the application of the peptide. The coloring scheme relates peptide labels to the corresponding curves. For the sake of visual clarity, the results from HT-1080 are distributed between panels A and B and the results from ZR-75-1 are distributed between panels C and D.

To evaluate the activity of DIL, CopYL and seven other peptides described in Table 1, we have conducted the dose-response measurements on the sensitive (HT-1080) and insensitive (ZR-75-1) cancer cell lines. The results are shown in Fig. 7A–D. For fibrosarcoma cells HT-1080, the best results are obtained using the peptide DOL with presumed disulfide oxidase properties. The MTS assay data indicate that cancer cells are essentially fully eliminated after 96 h incubation with 20 μM DOL. This is a much better performance than shown by GO-202 where the survival rate under identical conditions is 16%. Following DOL, the second best result is demonstrated by DIL. Furthermore, both CCC and GO-202-ox outperform the standard GO-202, although only by a small margin. Five other peptides turn out to be inefficient compared to GO-202; predictably, this group includes the control peptide SQS.

In the case of the breast carcinoma cells ZR-75-1 the absolute efficiency of all peptides is much lower. However, the best result still belongs to DOL – at the concentration 20 μM it is three times more effective in killing cancer cells than GO-202. In addition to DOL, two other peptides, DIL and CCC, significantly outperform GO-202. Finally, GO-202-ox shows small, but consistent improvement over GO-202. All other peptides should be viewed as inactive – they lag behind the modestly active GO-202.

The series of measurements illustrated in Fig. 7A–D allowed us to identify the three most potent peptides, DOL, DIL, and CCC, whose activity substantially exceeds that of the equivalent GO-202 peptide. As a next step, we set out to test the cytotoxic effect of these peptides on the normal cells. In addition to the primary culture of dermal fibroblasts, which has been discussed above, we have also used human endometrial mesenchymal stem cells (eMSC). The eMSC is a good example of normal somatic cells, which are characterized by multipotency and high rate of cell proliferation, but with limited number of cell divisions (i.e. unlike transformed cell lines, the eMSC are not immortal). The dose response curves obtained for these two normal cell cultures, Fig. 7E,F, lead to a number of interesting observations. (i) Not only dermal fibroblasts (cf. Fig. 2), but also eMSC turn out to be highly sensitive to the effect of GO-202 and the new cytotoxic peptides. (ii) Both DOL and CCC outperform GO-202, with DOL showing strongest effect in dermal fibroblasts and CCC showing strongest effect in eMSC. (iii) At the low dose of 5 μM most peptides tend to stimulate cell growth. In dermal fibroblasts, CCC, DIL and GO-202 all produce small increases in proliferative activity of the cells, while in eMSC DOL and DIL register similar increases. Of note, this stimulatory effect has only been observed in the normal cells, but not in the cancer cells (cf. Fig. 7A–D). Such biphasic dose response (low-dose stimulation/high-dose toxicity) has been observed in a number of cell culture experiments using different peptides^43^. It is generally assumed that small dose of a cytotoxic agent can elicit an overcompensation response leading to modest cell growth. (iv) DIL is clearly less cytotoxic than GO-202 in both dermal fibroblasts and eMSC. This is in contrast to the cancer cells, where DIL was consistently more cytotoxic than GO-202.

Let us generalize the observations from Fig. 7. We have found that 4 out of 5 peptides designed on a basis of their presumed redox properties turned out to be more potent than the equivalent GO-202 peptide. The only exception is the disulfide-reductase-like peptide DRL, which proved to be inactive. On the other hand, 3 peptides which have been designed with a different mechanism in mind all proved to be inactive.

The situation with the peptide DRL deserves a separate comment. It was modeled after the previously described thioredoxin-mimetic peptide NAc-CGPC-NH_2_^44^. To obtain a better insight into the action of DRL, it would be desirable to explore a longer sequence including flanking residues. For instance, an alternative design based on human thioredoxin could read [**R**]_9_TWCGPCKM (note the presence of both hydrophobic and positively charged residues in the flanking positions). Future investigation of this peptide should clarify whether the peptide modeled on disulfide reductase possess cytotoxic properties similar to other redox-active peptides investigated in this work. At this point we offer a general observation – that the peptides with designed *disulfide oxidoreductase* properties are typically more cytotoxic than the benchmark GO-202 peptide. The ramifications of this result for the mechanism of cytotoxicity are discussed in the next section.

Finally it should be pointed out that high-efficacy new peptides DOL and DIL do not bear any significant similarity to MUC1-CD. While GO-203 formally shares 7-residue fragment with MUC1-CD (although, in fact, there is a fundamental difference because the peptide consists of D-amino acids), both DOL and DIL have nothing in common with MUC1-CD except for a pair of proximal cysteines. It can be therefore confidently suggested that cytotoxic activity of these peptides is unrelated to MUC1, which supports our previous conclusions (see Fig. 2 and related discussion).

### Cytotoxic mechanism of CxC- and CxxC-containing peptides

**Figure 8:**
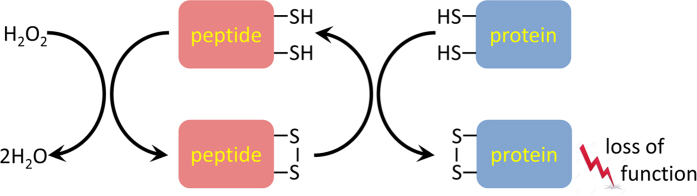
Proposed mechanism of cytotoxic activity of CxC- and CxxC-containing peptides. In addition to *intra*molecular disulfide bonds, the same mechanism can produce non-native *inter*molecular disulfide bridges between cytosolic proteins^45^. ROS molecules are represented by hydrogen peroxide, but in reality a number of different oxidants can be involved in this reaction.

Based on the above evidence we propose a mechanism of action for the original GO peptides as well as the new cytotoxic peptides described in the previous section. The presumed mechanism is schematically illustrated in Fig. 8 and briefly discussed below.

All but one peptides investigated in this study have been synthesized in the reduced form. However, when added to the cell culture media they are partially oxidized (see below for details). Once inside the cell, the peptides become a part of the cytosolic redox equilibrium, which is dominated by glutathione. Although cytosol is generally considered to be a reducing environment, there is a significant fraction of oxidized species. For example, in the healthy resting cells the proportion of oxidized glutathione is ca. 1%, whereas for the cells experiencing oxidative stress this proportion increases to 10% or even higher^46^. Likewise, it can be expected that a certain fraction of CxC- or CxxC-containing peptides is oxidized in the cytosol and form an internal disulfide, see Fig. 8.

The crux of our hypothesis is that the disulfide-bonded peptide can act as disulfide oxidase. In doing so, it interacts with various cytosolic proteins where it oxidizes cysteines and creates disulfide bridges. The corresponding disulfide-dithiol exchange reaction is illustrated in the right portion of Fig. 8. In this reaction the peptide itself becomes reduced; it is subsequently re-oxidized by low-molecular-weight oxidants and then the entire cycle is repeated. Thus, the peptide acts as a catalyst, inducing formation of disulfide bonds in cytosolic proteins. Those disulfide bonds are outside the range of norm, i.e. they should not exist under normal conditions or should be formed only infrequently. Consequently, they interfere with protein function; they may also destabilize the affected proteins, leading to increased proteolysis and/or aggregation^47^. All of this disrupts the normal operation of the cell machinery, causing apoptosis.

The above brief description requires some additional comments. Clearly the investigated CxC- or CxxC-containing peptides can only be weak mimetics of true disulfide oxidases. They should also lack the selectivity of the specialized enzymes. On the other hand, these peptides can meaningfully interact with a broad range of proteins and induce the formation of disulfide bridges with greater efficiency than can be expected from a direct attack by ROS. For instance, the presence of hydrophobic residues in the peptides DOL and DIL facilitates their (transient) binding to protein targets and thus increases the success rate of the disulfide-dithiol exchange reaction^41^. Therefore the cytotoxic CxC- and CxxC-containing peptides can be described as “amplifiers of ROS damage”. The cyclical nature of this process is especially important: one molecule of the peptide can oxidize multiple proteins before it is itself degraded or transported out of the cell.

Note that in addition to disulfide oxidase activity, CxC and CxxC peptides could in principle exhibit the reductase activity (cf. the emerging concept of reductive stress^48^). Indeed, our experimental results do not rule out such possibility (see previous section). If so, then one can envision the reverse mechanism, where the peptide is first reduced (e.g. by GSH) and then enters into dithiol-disulfide exchange reaction to break one of the protein’s native disulfide bonds. In the reducing cytosol environment, the reductase function of the CxC and CxxC peptides is likely secondary to the more important oxidase function. Nevertheless, in order to maintain generality, we refer to them as *disulfide*-*oxidoreductase*–like peptides or, in abbreviated form, DO peptides. It is assumed that the previously reported GO peptides constitute a small subset of the broader DO class.

At this point it is appropriate to briefly summarize the experimental evidence supporting the hypothetical mechanism Fig. 8. First, the reactive properties of thiols are obviously the key to the cytotoxic activity of the DO peptides. When cysteine residues are replaced with serines (small polar amino acids that are similar to cysteines, but lack thiol groups) the peptides’ cytotoxic activity is completely abrogated. Second, the design of new DO peptides strongly suggests that their activity stems from their redox properties. Third, GO-202 that has been prepared in the oxidized form shows small but systematic increase in activity compared to the reduced GO-202. This is consistent with our hypothesis – indeed, the oxidized peptide is “primed for action” in its role of disulfide oxidase (see Fig. 8) and therefore is expected to be somewhat more efficient. Fourth, GO-203 treatment leads to small but statistically significant drop in the intracellular ROS level in the sensitive cell line HT-1080. This is also in agreement with the described scenario where the peptide can initially reduce the amount of reactive oxygen species (cf. conversion of H_2_O_2_ to water illustrated in Fig. 8), but does not show any substantial antioxidant effect. Fifth, the onset of apoptosis in the treated cells is rapid, ca. 30 min after the application of GO-203. This is also consistent with the mechanism shown in Fig. 8. Indeed, it can be expected that the oxidative damage to cytosolic proteins occurs fairly quickly and is wide-spread. Some of the affected proteins are critical for cell survival – oxidative damage to these proteins should rapidly trigger the apoptosis. This situation can be contrasted with other cytotoxic agents. For example, the compounds that block cell division cycle do not induce apoptosis until many hours after the treatment^49^. Among these five observations, the first two carry more weight while the other three offer more of an indirect support to the stated hypothesis. Taken together these results provide substantial indication that the cytotoxic properties of DO peptides are indeed linked to their disulfide oxidoreductase activity.

### Validation of the proposed mechanism

**Figure 9:**
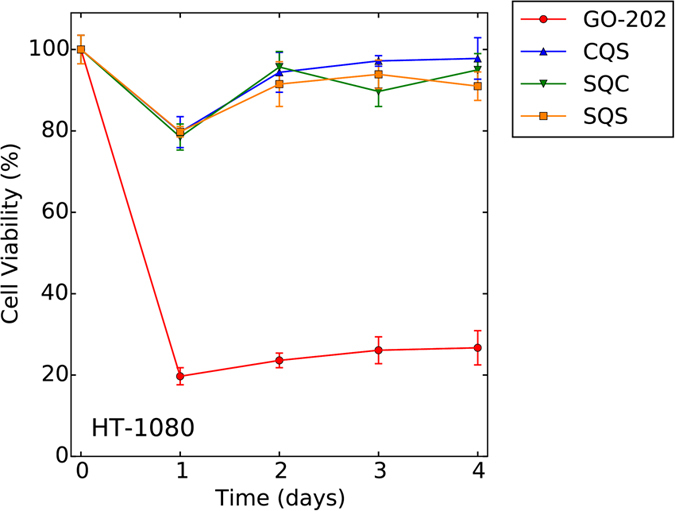
Viability of HT-1080 cells following single 20 μM dose of GO-202, SQS, CQS ([**R**]_9_CQSRRKN), or SQC ([**R**]_9_SQCRRKN) peptide. The presented MTS assay data confirm that cell death occurs soon after the GO-202 treatment. After sustaining the initial damage, the treated cells start to recover, showing slightly better dynamics than control cells.

In order to validate the mechanism described in the previous section, we have performed a number of additional experimental measurements. First, we have tested two peptides that have the same sequence as GO-202 but with one serine-for-cysteine substitution (termed CQS and SQC). We predicted that both variants should display a significantly reduced cytotoxic activity. This prediction has been fully confirmed. As shown in Fig. 9, both single-cysteine variants are inactive, similar to the ineffective SQS peptide. This observation supports the mechanism illustrated in Fig. 8, which is dependent on two cysteine residues.

We would like to note, however, that in general single-cysteine peptides may retain some degree of disulfide oxidoreductase activity. One example is glutathione, which contains a single thiol group. Upon oxidation, glutathione forms a disulfide-bonded dimer GSSG which is capable of disulfide-dithiol exchange reaction along the lines of Fig. 8. The disulfide isomerase activity of GSSG/GSH is well documented^50^,^51^. On a related note, it has been observed that a single-cysteine variant of protein disulfide isomerase (PDI) may remain moderately active^52^. Such PDI mutant first forms an intermolecular disulfide bridge with its substrate; the subsequent disulfide-thiol exchange reaction leads to formation of an intramolecular disulfide bridge within the substrate and release of the PDI. Therefore, one may generally expect to find a reduced level of activity in the single-cysteine variants of DO peptides, but not necessarily a complete loss of activity such as observed in this particular case, see Fig. 9.

**Figure 10:**
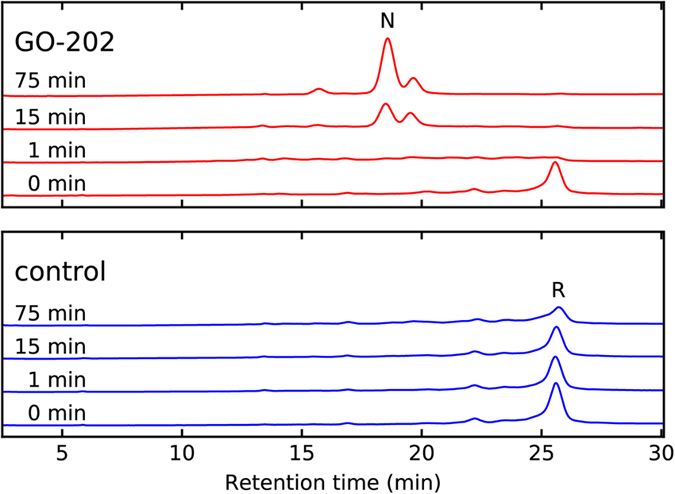
Oxidative refolding of hen egg lysozyme as monitored by reverse-phase HPLC. The elution positions of native and fully reduced denatured lysozyme are denoted by letters N and R, respectively. Top panel (red traces) illustrate protein renaturation in the presence of GO-202; bottom panel (blue traces) represent control measurements with no peptide. The reaction time *T*_*ox*_ ranging from 0 to 75 mins is listed on the left side of the individual chromatograms. Similar results have been obtained using GO-202-ox (not shown).

We have also conducted *in vitro* experiments to directly probe disulfide oxidoreductase properties of GO-202 peptide. For this purpose we have used the well-established lysozyme refolding assay^53^. In brief, GO-202 is applied to the sample of reduced and denatured hen lysozyme. The protein then undergoes oxidative refolding, assisted by GO-202 which plays the role of disulfide oxidase (isomerase). After a certain interval of time (*T*_*ox*_) this process is quenched by adding an aliquot of acid and the sample is loaded on the reverse phase column. The chromatography data, as shown in Fig. 10, allow one to monitor the progress of oxidative folding.

The lower panel in Fig. 10 shows the results of control experiment which omits GO-202. As can be seen from the graph, reduced and denatured lysozyme elutes with the retention time *t*_*ret*_ = 25.5 mins (marked in the chromatogram by the letter R). After the sample is kept on the bench for *T*_*ox*_ = 75 mins, this peak becomes considerably smaller; the intensity is redistributed to multiple minor peaks with *t*_*ret*_ between 13 and 25 mins corresponding to various non-native disulfide-bonded intermediates. Hence, as a result of exposure to oxygen of air lysozyme becomes partially oxidized after ca. 75 mins.

In the presence of GO-202 this process occurs much faster, see upper panel in Fig. 10. Indeed, already after *T*_*ox*_ = 1 min we no longer observe a distinct peak from reduced lysozyme. Instead, the signal comes from a diverse mixture of disulfide-bonded species. After 15 mins a significant fraction of lysozyme appears in its fully oxidized natively folded form (marked in the chromatogram by the letter N). Finally, after 75 mins this native form becomes dominant, with two additional partially folded intermediates visible in the chromatogram. This behavior firmly identifies GO-202 as moderately effective disulfide oxidoreductase. The obtained data are, in fact, similar to what has previously been observed for oxidative refolding of lysozyme in the presence of GSSG (refs 53 and 54; also measured by us to test the experimental protocol). These results provide direct and unequivocal evidence in support of the mechanism in Fig. 8.

There is one additional question that needs to be asked in relation to Fig. 10: what is the oxidation state of GO-202 before it is introduced into the system? Considering that GO-202 causes rapid (within 1 min) oxidation of lysozyme, one should assume that the initial peptide material is at least partially oxidized^50^. We have experimentally confirmed that this is indeed the case. As indicated in *Methods*, all peptides used in this study except GO-202-ox have been manufactured in the reduced form. However, after being dissolved in a neutral or basic buffer they undergo relatively fast oxidation.

Consider the protocol used to handle peptide material in our cell culture experiments. Briefly, the peptides were dissolved in PBS, aliquoted in Eppendorf tubes, and then immediately placed in −20 °C freezer for storage. At a later date they were retrieved from the freezer and thawed for several minutes before use. Even though this procedure is rather conservative, our analysis shows that it leads to substantial peptide oxidation. For example, we analyzed the sample of GO-202 that had remained frozen for two weeks. ^1^H NMR assay was employed to determine the overall concentration of the peptide, while the assay using Ellman’s reagent was employed to determine the content of free thiols (see *Methods*). As it turns out, the proportion of oxidized and reduced peptide in this particular sample was 1:3. Furthermore, the peptide is likely to undergo further oxidation after being dissolved in the medium with growing cells, which is known to contain a significant amount of ROS^55^ (see also Fig. S4).

Thus, the situation with Cx(x)C-containing peptides is similar to that with glutathione GSH/GSSG mixtures. It is well known that such GSH/GSSG mixtures are optimal to accomplish oxidative renaturation of target proteins^51^. The same effect is achieved by the mixture of oxidized and reduced Cx(x)C peptides, as demonstrated in Fig. 10. The realization concerning the oxidation status of peptide material also helps to interpret the results of cell viability experiments shown in Fig. 7B,D. As can be seen in these figures, GO-202-ox shows a slightly higher level of cytotoxicity than GO-202, but overall the dose-response characteristics for these two peptides are remarkably similar. This can be understood by noting that GO-202 becomes largely oxidized prior to entering the cells. This result is fully consistent with the proposed mechanism of peptide cytotoxicity and, in fact, provides further evidence in support of this mechanism.

The *in vitro* experiment Fig. 10 proves the ability of GO-202 to function as disulfide oxidoreductase. Obviously, in the cellular milieu the interactions of GO-202 should be far more complex than in this model experiment. It can be expected that inside the cells Cx(x)C peptides interact with a multitude of protein targets. It is likely, however, that cell death is triggered mainly through the interactions with several important targets that happen to be susceptible to oxidation by Cx(x)C peptides. The identity of these key targets currently remains unknown and it will not be a simple matter to identify them.

### Concluding remarks

Some interesting examples of peptides with presumed DO mechanism can also be found in the literature. For example, in their recent high-profile study of β-defensin 1, Schroeder *et al*. discovered a 7-residue peptide GKAKCCK with pronounced antimicrobial activity^56^. The efficiency of this peptide was critically dependent on the two contiguous cysteines: mutating these residues to alanines or serines completely abrogated its activity, whereas other alterations such as sequence reversal preserved the activity. Other peptide sequences containing distant cysteine residues showed no evidence of antimicrobial properties. Given the well-established connection between antimicrobial and anticancer peptides^57^,^58^, it is likely that the mechanism of GKAKCCK (and, in fact, the mechanism of β-defensin 1) is similar to the mechanism of the DO peptides.

What is the potential value, if any, of the DO peptides in the context of therapeutic applications? There are indications that current DO peptides can show modest degree of selectivity toward cancer cells. For instance, it appears that DIL causes more damage to cancer cells than normal cells; in particular, at lower doses it seems to be well-tolerated by the normal cells. However, this is clearly insufficient to become a basis for any pharmaceutical designs. The current variants of DO peptides are broadly cytotoxic and therefore ill-suited for use as pharmaceuticals.

The key to future development of the DO peptides is to confer onto them the ability to selectively target cancer cells (or other diseased cells). In principle this can be achieved by coupling DO peptides with different transduction domains that can selectively penetrate into neoplastic cells. Several naturally occurring sequences that possess this property are known^59^; also an impressive array of artificial tumor-homing peptides has been recently developed^36^,^60^. Although such targeting strategies have not yet translated to clinical tests, one may expect to see a rapid progress in this area. Despite a number of outstanding challenges, the global market for pharmaceutical peptides shows rapid and sustained growth^61^. This augurs well for future development of tumor-targeted peptides. We expect that DO peptides and their future targeted variants will become a useful addition to these broadly based efforts.

## Methods

### Cell culture

Human HT-1080 fibrosarcoma and A549 lung carcinoma cells (from Cell Culture Collection of vertebrates, Institute of Cytology, Russian Academy of Sciences) were cultured in DMEM supplemented with 10% FBS and 20 μg/ml gentamicin (Biolot, Russia). Human ZR-75-1 breast cancer cells (obtained from the same source) were grown in RPMI 1640 media supplemented with 10% FBS and 20 μg/ml gentamicin. Human endometrial mesenchymal stem cells (eMSC) were derived from a desquamated endometrium of menstrual blood from healthy donors as described previously^62^ eMSC were cultivated in DMEM/F12 supplemented with 10% FBS, 1% L-glutamine and 1% penicillin/streptomycin. eMSC from passages 9–13 have been used in the experiments. Human fibroblasts isolated from forearm skin of a healthy donor (kindly provided by Dr. N.M. Pleskach) were cultured in DMEM low glucose (1 g/l) supplemented with 10% FBS, 20 μg/ml gentamicin and 0.3 mg/ml L-glutamine. Fibroblasts from passages 4–9 have been used in the experiments. All cell lines were grown at 37 °C in a humidified atmosphere containing 5% CO_2_. All experiments have been approved by the Ethics Committee of the Pavlov Medical University (St. Petersburg, Russia) and conducted in accordance with the institutional guidelines. All cell donors signed an informed consent for voluntary participation.

### Peptides

The peptide CCC was designed by using the server http://birg4.fbb.utm.my/cmd to conduct searches among the triplets of amino acids RCx and xCR. Other peptides have been designed as discussed in the text. The peptides were synthesized by Pepmic Co. Ltd. (Suzhou, China) in the reduced form, except GO-202-ox which was intentionally manufactured in the oxidized form. The peptides were delivered lyophilized and then dissolved in PBS (pH 7.4). The peptide concentration in stock solutions was determined using ^1^H NMR (spectra collected at 500 MHz proton frequency). Briefly, the spectral signal from arginine methylenic protons at 3.2 ppm was used to determine the concentration of the peptide. A sample of L-arginine was used as an external reference while the water signal was used as an internal standard, producing identical results. The content of free thiols in the peptide samples was determined using Ellman’s reagent; the concentration of 2-nitro-5-thiobenzoate was quantified by measuring the absorbance at 412 nm.

### Cell viability

Cell survival was measured using CellTiter 96 AQueous One Solution Cell Proliferation Assay (MTS test, Promega, USA). Cells have been seeded in a 96-well flat-bottom plate in 100 μl of culture medium (10^3^ cells/well for A549, HT-1080, dermal fibroblasts and eMSC or 5 × 10^3^ cells/well for ZR-75-1) and allowed to attach for 24 h. The starting amount of cells was chosen to account for their growth rate and degree of spreading such as to avoid excessive dilution in the beginning of the experiment or control cell overgrowth toward the end. After 24 h, 10 μl of peptide stock solution or PBS (control) were added to the wells. After 96 h, 20 μl of the MTS-reagent were added to each well and 1–2 h later optical density was measured at the wavelength 492 nm using the 96-well plate reader (Titertek Multiskan, Labsystems, Finland). All experiments were conducted in duplicate or triplicate. The same protocol has been used with the other treatment schemes using multiple additions of the peptide, see Fig. 2, or different incubation times, see Fig. 9.

### Western Blot

Adherent cells were collected using cell scraper, washed with cold PBS and lysed in 50 μl of lysis buffer (0.1% SDS, 0.5% Triton X-100, 0.2% PMSF, and protease inhibitor cocktail (Sigma, USA) in PBS) on ice for 20 min. Electrophoretic separation of cell lysate proteins was performed in 10% SDS-PAGE gel along with molecular weight markers (Thermo Scientific, Lithuania). The separated proteins were transferred to a 0.45-μm nitrocellulose membrane (Bio-Rad, USA) and probed using Anti-MUC1-CD (AB2) rabbit polyclonal antibody (catalogue code AV41446, Sigma-Aldrich, USA) diluted 1:250 and Anti-β-actin mouse monoclonal antibody diluted 1:5000. Western blot signals were detected using secondary alkaline phosphatase-conjugated anti-rabbit and anti-mouse antibodies diluted 1:30000 and BCIP-NBT color development substrate (Bio-Rad, USA).

### Apoptosis and cell cycle assays

The adherent cells were rinsed with PBS, harvested using trypsin-EDTA solution and suspended in the growth medium. To analyze cell cycle phase distribution, 200 μg/ml of saponin (Fluka, USA), 250 μg/ml RNase A, and 50 μg/ml of propidium iodide (both Sigma-Aldrich, USA) were added to each sample tube, thus obtaining DNA stain buffer. After 1 h incubation at room temperature, the samples were analyzed by flow cytometry. To detect apoptosis by flow cytometry, CellEvent Caspase-3/7 Green Flow Cytometry Assay Kit (Thermo Fisher Scientific, USA) and Annexin-V FITC conjugate (BD Biosciences, USA) in combination with propidium iodide have been employed following the manufacturer’s protocols.

### ROS measurements

The cells were seeded into 35-mm Nunc culture dishes (Thermo Scientific, USA) at 1.2 × 10^5^ cells per dish and allowed to attach for 24 h. To monitor intracellular ROS in a time-dependent manner, the cells were incubated for 30 min with 10 μM of carboxy-H2DCFDA (Invitrogen, USA), then harvested with trypsin-EDTA and suspended in a fresh medium. After that GO-203 was added directly to the suspension and cell fluorescence was measured by flow cytometry at multiple time points during the following 2 hours. The same procedure was used for mitochondria-specific fluorescent dye Rhodamine 123 (Thermo Fisher Scientific, USA), which was added to a concentration of 5 μM. The complementary side scatter measurements were conducted using 488 nm laser light. In another series of experiments, we have used the related indicator of oxidative stress, H2DCFDA (Invitrogen, USA). The experiment started by adding GO-203 to the cell growth medium; then 10 min later 5 μM of H2DCFDA has been added. After 20 min incubations with H2DCFDA, the cells were harvested with trypsin-EDTA and suspended in a fresh medium. Cell fluorescence was then immediately measured by flow cytometry. The same procedure was followed for the ROS-insensitive (control) modification of the fluorescent probe, DCFDA.

### Flow cytometry

Cell fluorescence was measured using CytoFLEX (Beckman Coulter, USA) flow cytometer equipped with 3 lasers (405, 488 and 633 nm). Cells were gated on FSC/SSC to remove debris. Mean fluorescence intensity from 10,000 cells was acquired.

### Live cell microscopy

For live cell microscopy, HT-1080 cells were seeded into 35-mm сell imaging dish with cover glass bottom (Eppendorf, Germany) and allowed to attach for 24 h. The cell response to 5 μM GO-203 exposure was monitored using the Axio Observer. Z1 inverted microscope (Carl Zeiss Microimaging, Germany) equipped with the temperature-, humidity- and CO_2_-controlled chamber. Micrographs were acquired every 30 sec for an observation period of 5 h using 63×/1.40 Plan-Apochromat oil-immersion objective and AxioCam HRm camera. The acquisition control and image processing were carried out using AxioVision 4.8.2 software (Carl Zeiss Microimaging, Germany).

### Disulfide oxidoreductase activity assay

Hen egg white lysozyme (Amresco, USA) was reduced/denatured and lyophilized according to the protocol by van den Berg *et al*.^54^. It was then dissolved in buffer B1 (8 M urea, 100 mM Tris-HCl, 100 mM NaCl, 1 mM EDTA, pH 8.5) to obtain solution with 100 μM lysozyme concentration. Refolding was initiated by rapid dilution of 50 μL of this solution in 150 μL of buffer B2 (100 mM Tris-HCl, 100 mM NaCl, 1 mM EDTA, pH 8.5) containing the appropriate amount of redox agent (none for control experiment, 1.0 mM/0.2 mM GSH/GSSG for calibration experiment, 1 mM of GO-202 for disulfide oxidoreductase activity assay). The reactions were carried out at room temperature and quenched by addition of 10 μL of 2.5 M HCl to produce a pH of ca. 2. Buffers B1 and B2 were degassed prior to use by applying vacuum while stirred on magnetic stirrer for 30 min.

Reverse-phase chromatography data were obtained using Shimadzu Nexera HPLC with detection at 280 nm wavelength. The column used was Phenomenex Luna C-18 (5 μm particle size, 100 Å pore size, 150 mm length, 4.6 mm diameter); sample injection volume was 20 μL. HPLC solvent A was H_2_O/0.2% (v/v) trifluroacetic acid (TFA), solvent B was 90% CH_3_CN/10% H_2_O/0.2% TFA. Lysozyme was eluted using 30 min linear gradient from 35% to 45% of solvent B with a flow rate of 1 mL/min. Chromatograms from calibration GSH/GSSG experiments were in agreement with the previously reported data^54^,^63^.

### Statistical analysis

Statistical data treatment was performed using the program Origin 6.0 (Microcal Software, USA). The data are presented as the mean and the standard deviation. Student’s t-test was used to compare differences between the means (p < 0.05).

## Additional Information

**How to cite this article**: Tyuryaeva, I. I. *et al*. Origin of anti-tumor activity of the cysteine-containing GO peptides and further optimization of their cytotoxic properties. *Sci. Rep.* **7**, 40217; doi: 10.1038/srep40217 (2017).

**Publisher's note:** Springer Nature remains neutral with regard to jurisdictional claims in published maps and institutional affiliations.

## Supplementary Material

Supplementary Information

Supplementary Film S1

## Figures and Tables

**Table 1:** Additional peptides containing CxC or CxxC motifs that have been investigated in this work.

Peptide abbreviation	Sequence	Comments
*Design based on redox properties and motifs found in redox-control proteins*		
GO-202-ox	[**R**]_9_C^*^QC^*^RRKN	Cysteines are disulfide-bonded
DOL	[**R**]_9_FFCPHCYQ	Based on the sequence of disulfide oxidase DsbA, powerful oxidizing enzyme^37^.
DIL	[**R**]_9_KCGCFF	Based on the previously described peptide with the properties of disulfide isomerase^41^.
DRL	[**R**]_9_GCGPCG	Based on the previously described peptide derived from disulfide reductase thioredoxin^44^.
CCC	[**R**]_9_CCCRRKN	Modification of GO-202 with C-for-Q substitution. According to PDB and UniProt analyses^64^, RCxCR sequence with x = C has highest propensity to form disulfide bridges (via the flanking cysteines).
*Design based on other known protein motifs*		
CopYL	[**R**]_9_IECNCIPGQCECKK	Based on copper-binding motif from bacterial CopY repressor^42^.
CXCL	[**R**]_9_GRCSCIS	Based on the sequence of human chemokine CXCL9^65^.
TAT-CQC	[**YGRKKRRQRRR**]CQCRRKN	Modification of GO-202 where poly-arginine tag is replaced with HIV-1 TAT transduction domain^66^.
*Control*		
SQS	[**R**]_9_SQSRRKN	Modification of GO-202 with S-for-C substitution
