# Supplementary material for: Origin of anti-tumor activity of the cysteine-containing GO peptides and further optimization of their cytotoxic properties
Source: Sci Rep. 2017 Jan 16;7:40217. doi: 10.1038/srep40217 (PMC5238392; doi:10.1038/srep40217)
Supplement: Supplementary Information [file srep40217-s1.pdf]

# Origin of anti-tumor activity of the cysteine-containing GO peptides and further optimization of their cytotoxic properties.

Irina I. Tyuryaeva,[1,2] Olga G. Lyublinskaya,[3] Ivan S. Podkorytov,[2] Nikolai R. Skrynnikov[2,4*]

[1] Institute of Cytology, Russian Academy of Sciences, St. Petersburg 194064, Russia
[2] Laboratory of Biomolecular NMR, St. Petersburg State University, St. Petersburg 199034, Russia
[3] Department of Intracellular Signaling and Transport, Institute of Cytology, Russian Academy of Sciences, St. Petersburg 194064, Russia
[4] Department of Chemistry, Purdue University, West Lafayette IN 47907, USA

[*] Corresponding author. E-mail: nikolai@purdue.edu

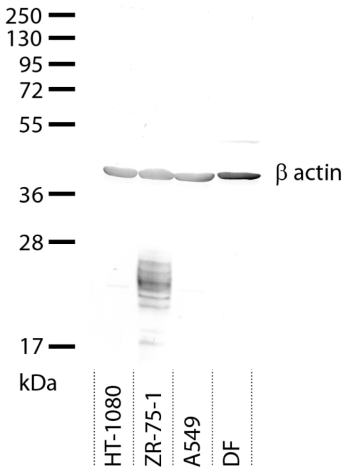


**Figure S1.** Low-exposure image of the Western blot shown in Fig. 1.

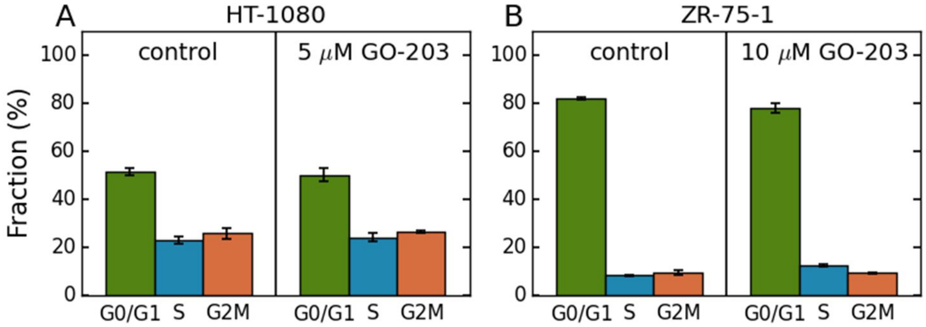


**Figure S2**. Cell cycle distribution for (A) HT-1080 cells treated with one-time dose of 5 μM GO-203 and (B) ZR-75-1 cells treated with one-time dose of 10 μM GO-203. The peptide concentrations have been chosen such as to induce significant cytotoxic effect without causing massive cell death. The data were taken 24 h after the application of the peptide.

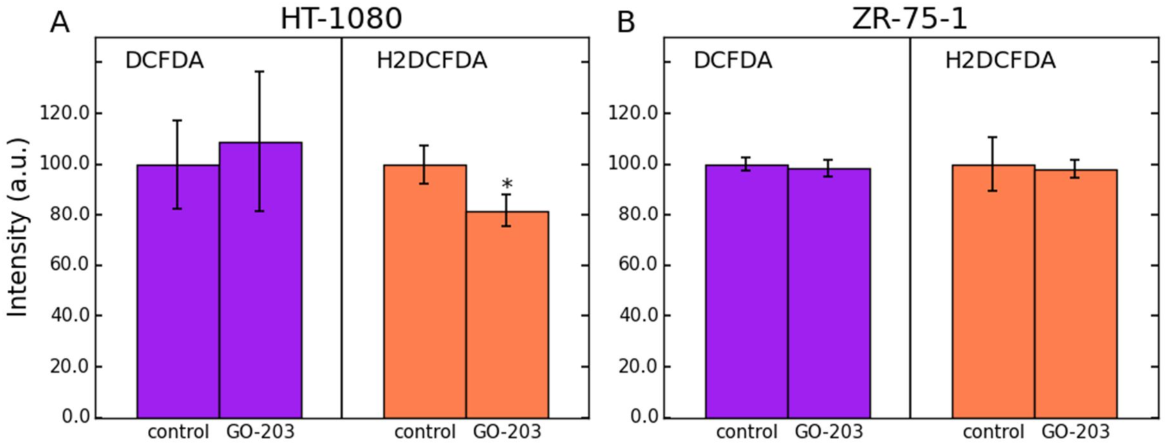


**Figure S3**. H2DCFDA/DCFDA experiment to assess the level of ROS in (A) HT-1080 cells after 30 min incubation with 5 μM GO-203 and (B) ZR-75-1 cells after 30 min incubation with 10 μM GO-203. A statistically significant difference in the intensity of DCF fluorescence response is marked by an asterisk.

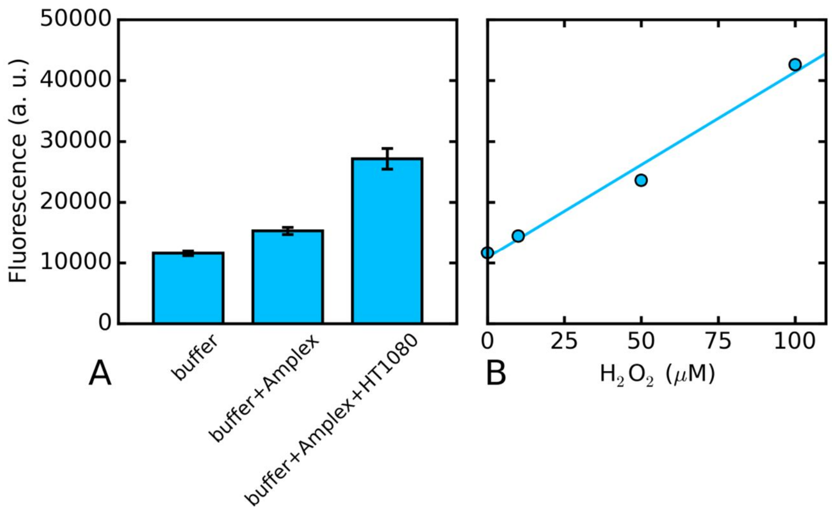


**Figure S4**. Amplex Red assay to quantify $H_2O_2$ efflux from HT-1080 cells (excitation 570 nm, detection 615 nm). (A) Left column: aliquot of the buffer solution as supplied by assay kit manufacturer (Invitrogen, USA). Middle column: aliquot of the buffer solution containing Amplex Red (incubated in the dark for 45 min at 37 °C). Right column: aliquot of the buffer solution containing Amplex Red incubated with HT-1080 cells. To obtain the latter sample, growth medium was removed from the dish containing $4.5\times10^5$ HT-1080 cells and replaced with the buffer solution containing Amplex Red. The cells were then incubated in the dark for 45 mins at 37 °C, and an aliquot of the solution was transferred to flat-bottom plate for analyses. (B) Calibration plot for detection of $H_2O_2$ in the buffer solution with Amplex Red. The results in panels (A) and (B) suggest that in 45 mins the cells secreted ca. 50 μM $H_2O_2$. This experiment demonstrates how cells can contribute to the ROS content in culture medium (see also Uy *et al.*, *J. Biomol. Tech.* **22**, 95-107 (2011)).

**Film S1**. Time-lapse microscopy video of HT-1080 cells following the addition of 5 μM GO-203. The first frame of the video is taken 7 min after the image Fig. 5a, which shows the same cells before the addition of the peptide. The video covers 4 h 20 min interval (the timer is displayed in the upper left corner of the screen).
